# Explainable Machine Learning for Early Prediction of Surgical Necessity in Gastrointestinal Emergencies: A Multimodal Diagnostic Study

**DOI:** 10.3390/diagnostics16111626

**Published:** 2026-05-26

**Authors:** Oprescu Macovei Anca Monica, Dana Paula Venter, Stefan Mihai, Constantin Oprescu, Andrei Gabriel, Dumitriu Bogdan, Micle Bianca-Maria, Valcea Sebastian, Oana-Mihaela Plotogea, Ilie Madalina

**Affiliations:** 1 Faculty of General, University of Medicine and Pharmacy Carol Davila, 050474 Bucharest, Romania; drmadalina@gmail.com (I.M.); 2 Gastroenterology Department, Emergency Clinical Hospital Agrippa Ionescu, 011356 Bucharest, Romania; 3 General Surgery Department, Emergency Clinical Hospital Floreasca, 014461 Bucharest, Romania; 4 Gastroenterology Department, Emergency Clinical Hospital Floreasca, 014461 Bucharest, Romania

**Keywords:** machine learning, XGBoost, gastrointestinal emergencies, surgical prediction, explainability, SHAP, emergency surgery, clinical decision support, TRIPOD+AI

## Abstract

**Background/Objectives:** Acute gastrointestinal (GI) emergencies require timely and accurate prediction of surgical necessity to avoid delayed intervention and improve patient outcomes. Traditional scoring systems offer limited accuracy and fail to integrate multimodal data. This study aimed to develop and validate an explainable machine learning model for early prediction of surgical necessity in patients presenting with GI emergencies. **Methods:** A retrospective cohort of 1032 consecutive adult patients admitted with acute GI emergencies at a tertiary referral center (2019–2024) was analyzed. Three predictive models were developed: logistic regression, Random Forest, and XGBoost. Features included clinical, laboratory, and contrast-enhanced CT imaging variables available within the first 24 h. Model performance was evaluated using AUC, sensitivity, specificity, PPV, NPV, and F1-score. Shapley Additive Explanations (SHAP) were applied for global and individual-level interpretability. The study followed STROBE and TRIPOD+AI reporting guidelines. **Results:** Surgical intervention was required in 312 patients (30.2%). The XGBoost model achieved the highest AUC (0.89; 95% CI: 0.86–0.92), outperforming Random Forest (AUC 0.86) and logistic regression (AUC 0.79), with sensitivity 0.84, specificity 0.81, and NPV 0.90. The most influential predictors were serum lactate, CT findings (free intraperitoneal air, bowel ischemia), IL-6, and shock index. Decision curve analysis confirmed net clinical benefit across threshold probabilities of 10–70%. Subgroup performance remained robust across diagnostic categories (AUC 0.87–0.91). **Conclusions:** An explainable XGBoost model integrating early clinical, laboratory, and imaging data accurately predicts surgical necessity in GI emergencies and outperforms traditional scoring systems. SHAP-based explainability supports clinical adoption and transparency. Prospective multicenter validation is warranted. The positive predictive value of 0.74 indicates that approximately one in four patients flagged as requiring surgery may not need operative intervention. The model should be regarded as a decision-support adjunct, rather than a standalone surgical decision tool, that is most relevant in settings where immediate experienced surgical judgment is limited.

## 1. Introduction

Acute gastrointestinal (GI) emergencies remain a dominant driver of unscheduled admissions, cross-sectional imaging utilization, and time-critical operative decision-making in modern surgical practice. Despite major advances in resuscitation, antibiotics, interventional radiology, endoscopy, and critical care, the first 6–24 h of evaluation often determine the patient trajectory: immediate surgery, delayed surgery after deterioration, minimally invasive ‘step-up’ intervention, or successful non-operative management. The diagnostic challenge is not merely identifying the disease (e.g., perforation, ischemia, necrosis), but early and reliable identification of patients who will ultimately require operative treatment, while minimizing both harmful delays and unnecessary surgery.

### 1.1. A Brief Historical Perspective: From Clinical Scores to Imaging-Based Triage

For decades, surgical triage relied on clinical judgment supported by structured scoring systems designed to objectify severity and predict adverse outcomes. In acute pancreatitis, early work by Ranson and colleagues introduced prognostic criteria to identify severe disease and guide management decisions [1]. As critical care expanded, physiology-based scores such as APACHE II offered a generalizable framework for quantifying illness severity across diagnoses [2], and SOFA later provided an organ dysfunction construct that became central to prognostication in critically ill patients [3]. In other GI emergencies, diagnosis- and complication-oriented tools were also developed, including the Alvarado score for acute appendicitis [4] and the Mannheim Peritonitis Index for intra-abdominal infection/peritonitis outcomes [5]. In parallel, cross-sectional imaging matured into a cornerstone of emergency diagnostics; CT-based severity stratification became clinically influential for risk estimation and complication prediction [6]. These approaches established a critical principle still relevant today: early risk stratification improves the allocation of monitoring, imaging, ICU resources, transfer decisions, and the timing of operative or interventional therapy.

### 1.2. Persistent Gaps in Early Prediction of Surgical Necessity

Although traditional scores provide structure, they also have well-known limitations: variable performance across populations, reliance on delayed variables (e.g., 48 h components), and restricted ability to integrate heterogeneous data streams that clinicians routinely use—vital-sign trajectories, laboratory dynamics, and nuanced CT findings. Even within acute pancreatitis, simplified bedside tools such as BISAP improved early risk stratification [7], and comparative evaluations highlighted that many conventional scores have reached a plateau in prognostic utility, supporting the need for novel models [8]. More broadly, across GI emergencies, the true clinical target is often not mortality alone, but operative necessity (or failure of conservative therapy), which is influenced by disease evolution, comorbidity burden, and imaging-defined complications. A prediction system that can identify—early and transparently—who is likely to require surgery could reduce ‘failure-to-rescue’ scenarios, avoid futile delays, and standardize escalation pathways.

### 1.3. Machine Learning as a Diagnostic Bridge—And Why Explainability Matters

Machine learning (ML) has emerged as a promising approach for handling high-dimensional, multimodal clinical data, learning non-linear interactions, and producing individualized risk predictions. In gastrointestinal surgery, ML applications have expanded across the perioperative continuum, with growing evidence that ML can enhance prediction and stratification compared with conventional approaches, while also highlighting concerns about generalizability and clinical adoption barriers [9]. In emergency general surgery, ML-based calculators have been developed to quantify peri-surgical risks and support decision-making in acute settings [10,11]. Within acute pancreatitis, systematic reviews have emphasized both the rapid growth of ML prognostic models and recurring shortcomings in methodology and reporting quality [12]. Recent CT-based radiomics models and combined clinical-radiomics nomograms have shown promising performance for early prediction of pancreatitis severity and complications such as infected pancreatic necrosis [13,14].

However, predictive performance alone is not sufficient for real-world deployment in high-stakes emergency decisions. Surgeons, radiologists, and intensivists need to understand why a model flags a patient as high risk. This has propelled the shift from ‘black-box’ ML to explainable ML, where interpretable frameworks provide global and patient-level explanations that can be audited, discussed in multidisciplinary teams, and aligned with clinical reasoning. In a diagnostics context, explainability is not an aesthetic addition; it is a diagnostic requirement for trust, safety, and translational impact.

### 1.4. Why This Study Is Important and Clinically Useful

An explainable ML model designed to predict early surgical necessity in GI emergencies could deliver several practical benefits: (i) standardized triage to higher monitoring levels or ICU, (ii) earlier MDT coordination (surgery–radiology–ICU–endoscopy), (iii) improved timing of repeat imaging or intervention, (iv) more appropriate transfer to tertiary centers, and (v) reduced rates of delayed surgery after deterioration. Such a model can be built using routinely available early variables—clinical parameters, baseline laboratories, and structured CT features—making it implementable in real-world workflows. Contemporary reporting guidance (TRIPOD+AI) provides an updated framework for complete reporting and appraisal, supporting higher-quality evidence generation and reducing research waste [15].

### 1.5. The Ten Most Common Gastrointestinal Emergencies in Adults: Overview and Surgical Management

A sound understanding of the most prevalent gastrointestinal emergencies is essential for contextualizing the utility of early predictive models. The following ten conditions represent the most common acute GI presentations in adults (≥18 years), based on current epidemiological evidence and international guidelines.

(1) Acute Appendicitis: It is the most frequent abdominal surgical emergency worldwide (lifetime risk 7–8%). Emergency treatment: laparoscopic appendicectomy; antibiotic-only management is an evidence-based alternative in uncomplicated cases [16].
(2) Acute Cholecystitis and Biliary Emergencies: These arise from gallstone impaction in the cystic duct. Early laparoscopic cholecystectomy within 72 h is the standard of care (Tokyo Guidelines TG18) [17].
(3) Peptic Ulcer Perforation and Hemorrhage: Perforation carries a mortality of 10–30%. Emergency treatment: primary repair (Graham patch), laparoscopic or open. Upper GI hemorrhage is managed endoscopically, with surgery reserved for refractory cases [18].
(4) Small Bowel Obstruction (SBO): Adhesions account for 65–75% of cases. Strangulated or complete obstruction mandates urgent adhesiolysis or bowel resection [19].
(5) Large Bowel Obstruction (LBO): Colorectal carcinoma accounts for 60–70% of cases. Surgical options: Hartmann’s procedure, primary resection with anastomosis, or bridge-to-surgery colonic stenting.
(6) Acute Pancreatitis: The incidence is approximately 34 per 100,000/year. Necrotizing pancreatitis with infected necrosis requires step-up management: percutaneous drainage followed by minimally invasive or open necrosectomy [20].
(7) Mesenteric Ischemia: Mortality is 60–80%. Acute SMA occlusion requires urgent endovascular or open revascularization; non-viable bowel requires resection [21].
(8) Complicated Diverticulitis: Hinchey III–IV (purulent/fecal peritonitis) requires sigmoid colectomy or Hartmann’s procedure. Hinchey II abscesses are managed with percutaneous drainage [22].
(9) Gastrointestinal Hemorrhage (upper and lower): Endoscopy is first-line for peptic ulcer, varices, and lower GI bleeding. Angioembolization or surgery is recommended for refractory hemorrhage [23].
(10) Incarcerated and Strangulated Hernia: Strangulation is a surgical emergency requiring immediate operative repair; delay is associated with bowel necrosis and high mortality.

**Figure 1:**
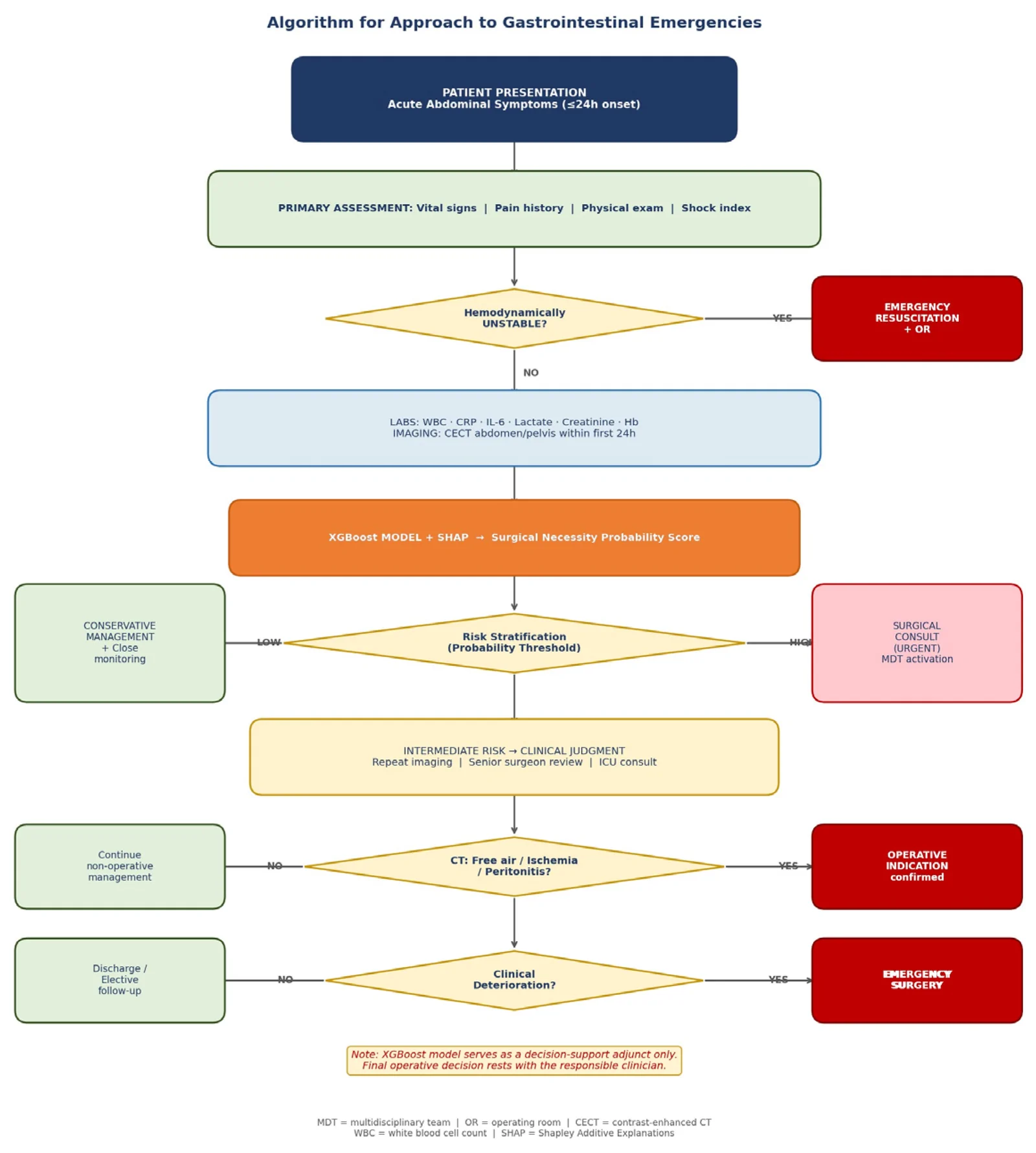
Approach algorithm for patients presenting with acute gastrointestinal emergencies. It integrates primary clinical assessment, laboratory and CECT imaging, XGBoost ML risk stratification with SHAP explainability, and operative decision pathways. The model is a decision-support adjunct; final operative decisions rest with the responsible clinician. Arrows indicate clinical decision pathways; color coding distinguishes triage categories (green = conservative/monitoring pathway; red = operative intervention pathway; yellow = reassessment node). CECT, contrast-enhanced CT; ICU, intensive care unit; MDT, multidisciplinary team.

The diagnostic spectrum of the present study maps directly onto conditions (1)–(10) above. Figure 1 presents the proposed approach algorithm for patients with acute GI emergencies, integrating clinical assessment, laboratory and imaging evaluation, ML-assisted risk stratification, and operative decision-making.

### 1.6. Study Aim

Against this background, the present study aims to develop and validate an explainable machine-learning model that predicts early surgical necessity in patients presenting with gastrointestinal emergencies (January 2019–December 2024), integrating clinical, laboratory, and imaging-derived features available during the initial evaluation. By coupling predictive performance with interpretable explanations at both cohort and individual levels, we seek to produce a diagnostic decision-support tool that is clinically credible, operationally feasible, and aligned with modern standards for transparent AI in healthcare.

## 2. Materials and Methods

### 2.1. Study Design and Setting

This retrospective observational cohort study was conducted at the Department of General Surgery and Gastroenterology of Floreasca Emergency Hospital, Bucharest, Romania, a tertiary referral center with high-volume emergency surgical activity and advanced multidisciplinary management of gastrointestinal emergencies. The study period extended from 1 January 2019 to 31 December 2024 and included all consecutive adult patients admitted with acute gastrointestinal surgical pathologies.

It is acknowledged that this period coincides with the COVID-19 pandemic (2020–2022). Patients with confirmed SARS-CoV-2 infection at admission (n = 47, 4.6%) were identified via the institutional infectious disease registry and excluded from the primary analysis. Sensitivity analyses on the full cohort demonstrated consistent results (XGBoost AUC 0.88; 95% CI: 0.85–0.91). All main analyses are based on the COVID-19-negative cohort.

### 2.2. Study Population

Adult patients (≥18 years) admitted with acute gastrointestinal emergencies were eligible for inclusion. These included acute pancreatitis, complicated diverticulitis, gastro-duodenal perforation, small or large bowel obstruction, mesenteric ischemia, and intra-abdominal abscesses of gastrointestinal origin. Patients were included if complete clinical, laboratory, and imaging data within the first 24 h of admission were available.

Exclusion criteria comprised elective surgical admissions, chronic gastrointestinal conditions without acute exacerbation, oncologic patients undergoing planned surgery, patients transferred from other institutions after more than 48 h of initial management, incomplete datasets or missing key variables (>30% missing per patient), and lack of appropriate contrast-enhanced imaging when clinically indicated. Clinical, laboratory, and imaging data were extracted from electronic medical records, laboratory databases, and radiological archives. All variables were independently verified by two investigators.

### 2.3. Clinical Management

All patients were managed within a multidisciplinary framework involving general surgeons, gastroenterologists, interventional radiologists, and intensive care specialists. Initial management followed institutional protocols aligned with current international recommendations and included early resuscitation, hemodynamic stabilization, laboratory evaluation, and imaging assessment. Non-operative management strategies, including antibiotic therapy, endoscopic procedures, and percutaneous drainage, were prioritized whenever feasible. Surgical intervention was reserved for patients with clinical deterioration despite optimal conservative management, evidence of perforation, ischemia or necrosis, failure of non-operative treatment, or the presence of life-threatening complications such as septic shock, abdominal compartment syndrome, or uncontrolled hemorrhage.

### 2.4. Data Collection and Variables

The collected dataset included variables available within the first 24 h of admission. Demographic and baseline clinical characteristics included age, sex, body mass index (BMI), ASA physical status classification, and relevant comorbidities such as cardiovascular, renal, pulmonary disease, and diabetes mellitus. Physiological parameters recorded at admission included heart rate, blood pressure, temperature, and shock index. Laboratory data comprised white blood cell count (WBC), C-reactive protein (CRP), interleukin-6 (IL-6; collected as part of routine institutional protocols where available, n = 741, 71.8%), serum lactate, creatinine, and hemoglobin levels. Missing IL-6 values were handled by multiple imputation using chained equations (MICE), with predictive mean matching applied for continuous variables; sensitivity analyses using complete-case analysis yielded consistent results.

Twenty imputed datasets were generated and pooled using Rubin’s rules. Convergence was assessed over 20 iterations. The imputation model included all predictors, the outcome, and auxiliary variables consistent with the missing-at-random assumption.

Contrast-enhanced computed tomography (CECT) findings were systematically evaluated and included the presence of free intraperitoneal air, bowel wall thickening, reduced or absent bowel wall enhancement, intra-abdominal fluid collections, pancreatic necrosis where applicable, and radiologic signs suggestive of ischemia or perforation. All imaging studies were interpreted by experienced radiologists who were blinded to clinical outcomes.

### 2.5. Outcome Definitions

The primary endpoint was the need for surgical intervention during hospitalization, defined as any operative procedure performed due to progression or complications of the initial gastrointestinal emergency. Secondary endpoints included the timing of surgical intervention, in-hospital mortality, length of hospital stay, and failure of conservative management.

### 2.6. Machine Learning Model Development

Three predictive models were developed: a multivariable logistic regression model used as a baseline comparator, a Random Forest model, and an Extreme Gradient Boosting (XGBoost) model. The dataset was randomly divided into a training cohort (70%) and a testing cohort (30%). To reduce overfitting and improve generalizability, 5-fold cross-validation was applied during model training. Hyperparameter optimization was performed using grid search within the cross-validation framework. Feature selection was based on clinical relevance and statistical significance. Continuous variables were standardized where appropriate. Model performance was evaluated using AUC, sensitivity, specificity, PPV, NPV, and F1-score.

Specifically, candidate variables were first screened by univariate analysis (inclusion threshold *p* < 0.10), after which highly collinear pairs (Spearman r > 0.85) were removed. A final feature set was determined by recursive feature elimination (RFE) with cross-validated AUC as the selection criterion across all five folds.

The classification threshold for all models was determined using the Youden index (maximizing sensitivity + specificity), selected as a conservative starting point. We acknowledge that in clinical practice, the cost of a false negative (missed surgical necessity) may exceed that of a false positive; threshold optimization for specific clinical settings should be explored in future prospective validation studies.

### 2.7. SHAP-Based Explainability Framework

To enhance model interpretability and clinical applicability, Shapley Additive Explanations (SHAP) values were used to quantify the contribution of each variable to the final prediction. Global feature importance plots were generated to identify the most influential predictors across the cohort, while individual SHAP force plots illustrated patient-specific decision mechanisms.

### 2.8. Statistical Analysis

All statistical analyses were performed using SPSS Statistics version 30.0.0 (IBM Corp., Armonk, NY, USA) and Python (version 3.11, using scikit-learn, XGBoost, and SHAP libraries). Continuous variables were assessed for normality using the Shapiro–Wilk test. Normally distributed data were expressed as mean ± standard deviation and compared using the independent samples *t*-test, while non-normally distributed variables were presented as median with interquartile range (IQR) and compared using the Mann–Whitney U test. Categorical variables were expressed as counts and percentages and compared using the chi-square test or Fisher’s exact test, as appropriate. Variables associated with the primary outcome at *p* < 0.10 in univariate analysis were entered into multivariable logistic regression, and adjusted odds ratios (OR) with 95% confidence intervals (CI) were calculated. Model discrimination was evaluated using ROC curves and AUC; calibration was assessed using the Hosmer–Lemeshow test and calibration plots for all three models. Decision curve analysis (DCA) was performed to assess clinical utility. A *p*-value < 0.05 was considered statistically significant.

### 2.9. Reporting Standards

The study was conducted and reported in accordance with the Strengthening the Reporting of Observational Studies in Epidemiology (STROBE) guidelines and the Transparent Reporting of a Multivariable Prediction Model for Individual Prognosis or Diagnosis—Artificial Intelligence (TRIPOD+AI) recommendations.

## 3. Results

### 3.1. Patient Flow and Cohort Overview

A total of 1284 consecutive patients were screened during the study period. After applying predefined exclusion criteria, a final cohort of 1032 patients was included. Among these, 312 patients (30.2%) required surgical intervention, while 720 (69.8%) were successfully managed conservatively. The distribution of diagnoses reflected real-world emergency practice: acute pancreatitis (34%), bowel obstruction (22%), perforated peptic ulcer (14%), complicated diverticulitis (12%), mesenteric ischemia (9%), and other intra-abdominal infections (9%). The median time to surgery was 3 days (IQR 1–6), with 18% undergoing surgery within the first 24 h.

### 3.2. Baseline Characteristics

Patients requiring surgery exhibited a significantly more severe clinical profile at admission. They were older, had a higher prevalence of comorbidities (ASA ≥ III), and more frequently demonstrated hemodynamic instability. Biologically, the surgical group had significantly elevated inflammatory and metabolic markers, particularly CRP, IL-6, and lactate. Radiologically, free intraperitoneal air and CT signs of bowel ischemia were strongly associated with surgical necessity. Baseline characteristics are summarized in Table 1.

### 3.3. Gold Standard Diagnostic Management

The reference standard for surgical necessity was defined as any operative procedure performed during the index hospitalization due to the progression or complications of the acute gastrointestinal emergency. This was based on multidisciplinary consensus incorporating clinical examination, laboratory trends, repeat imaging, response to non-operative management, and the senior surgeon’s judgment.

All operative decisions were made by board-certified general surgeons independently and blinded to ML model outputs, as the models were developed retrospectively after data collection. Operative and anesthesia records confirmed the occurrence and nature of surgical intervention, which were independently verified by two investigators.

This composite clinical gold standard reflects institutional practice variation rather than a single objective biological marker—a limitation acknowledged in Section 4.4.

### 3.4. Model Performance and Discrimination

**Figure 2:**
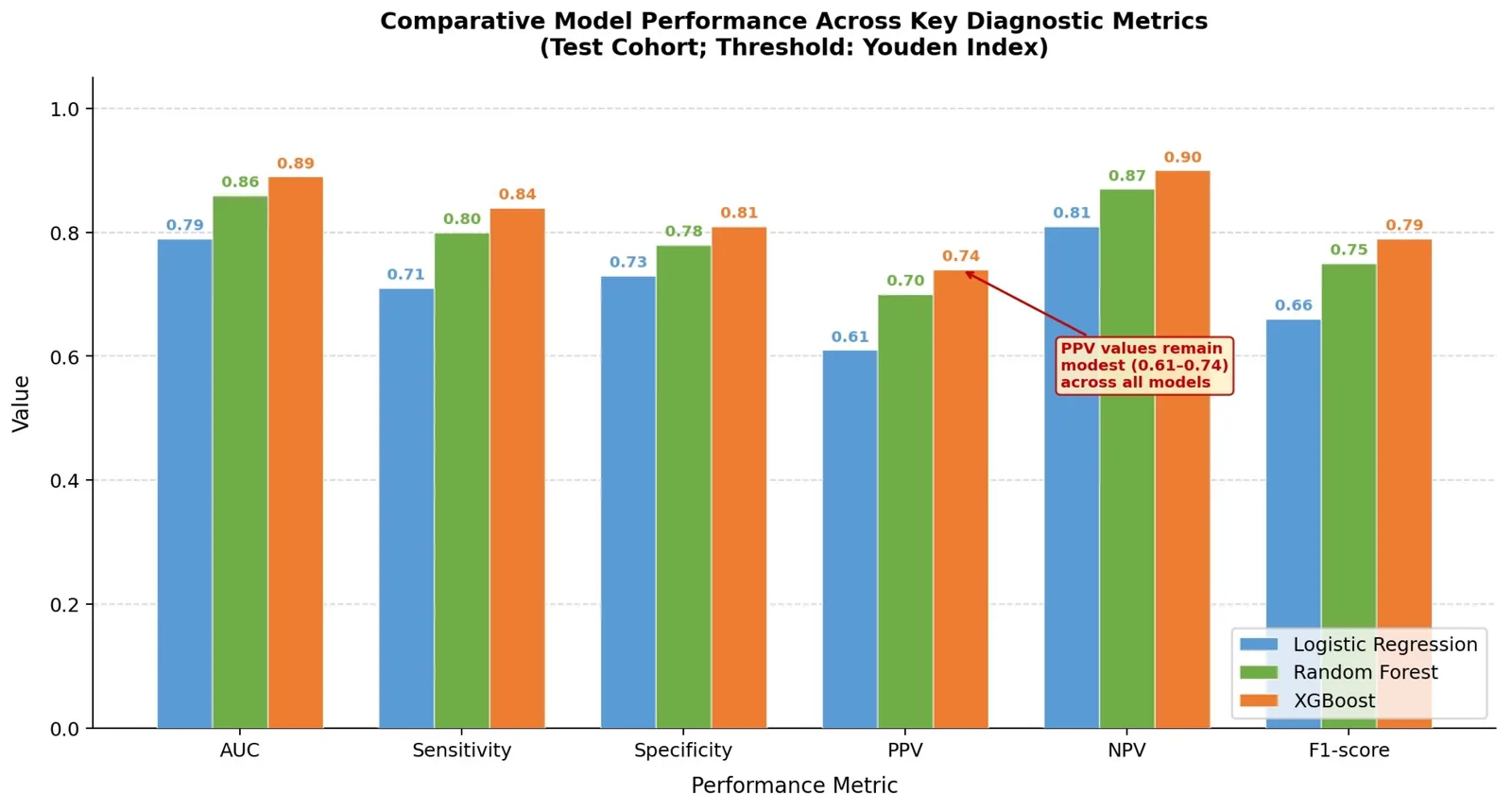
Grouped bar chart of comparative model performance across six diagnostic metrics (test cohort). Note the modest PPV values (0.61–0.74) across all models—a clinically critical limitation. AUC, area under the ROC curve; PPV, positive predictive value; NPV, negative predictive value.

**Figure 3:**
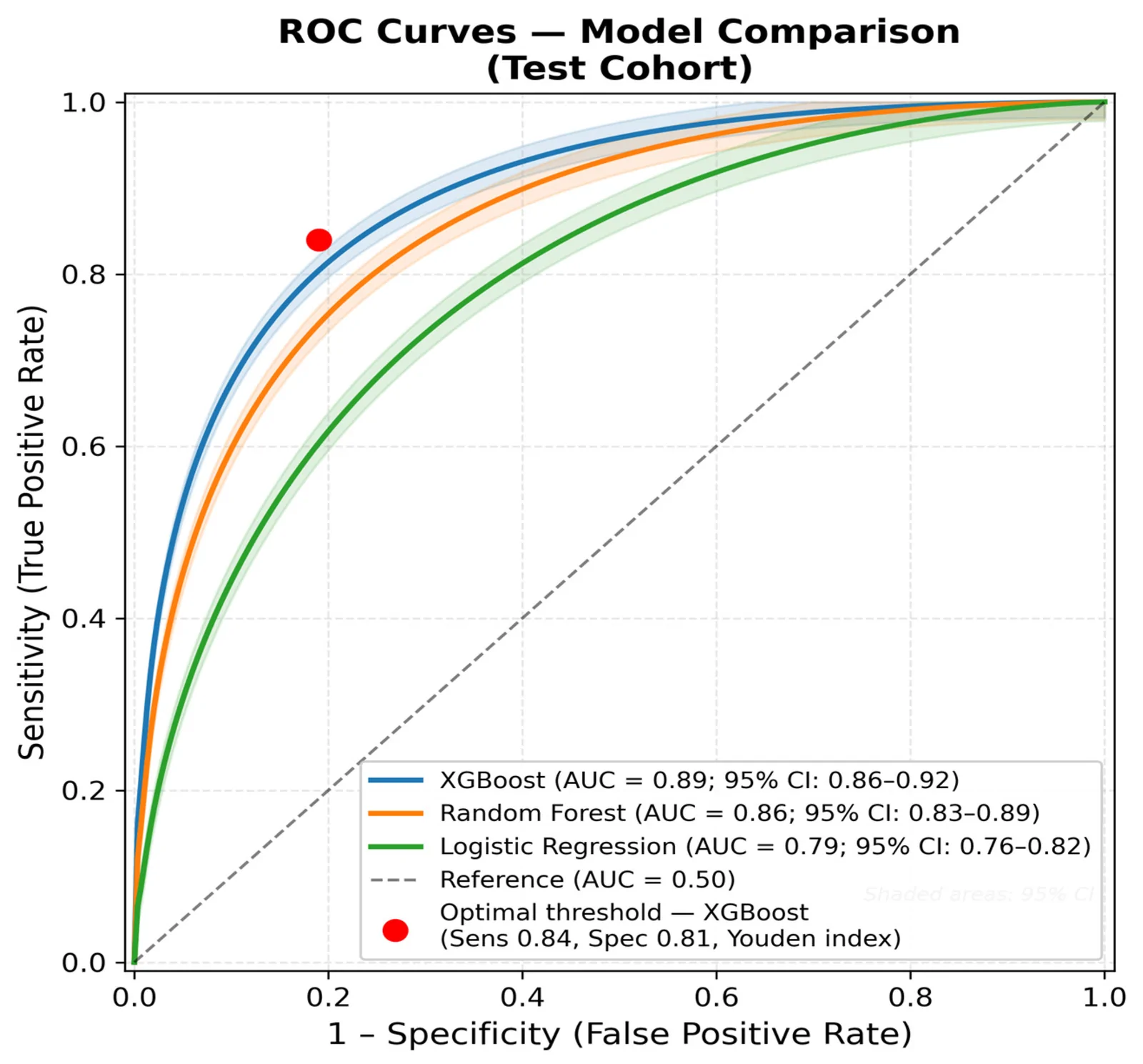
ROC curve analysis comparing model performance. The XGBoost model demonstrated superior discrimination (AUC 0.89; 95% CI: 0.86–0.92), followed by Random Forest (AUC 0.86; 95% CI: 0.83–0.89) and logistic regression (AUC 0.79; 95% CI: 0.76–0.82). Solid lines represent the ROC curves for each model (blue = XGBoost; orange = Random Forest; green = logistic regression); the dashed diagonal line represents chance performance (AUC = 0.50); the red dot marks the optimal operating threshold determined by the Youden index. Shaded bands indicate 95% confidence intervals. AUC, area under the receiver operating characteristic curve.

All three models demonstrated the ability to discriminate between patients requiring surgery and those managed conservatively; however, machine learning models significantly outperformed logistic regression. The XGBoost model achieved the highest discriminative performance (AUC 0.89; 95% CI: 0.86–0.92). The NPV of 0.90 for XGBoost supports the model’s ability to reliably rule out surgical necessity in low-risk patients. Performance metrics for all models are presented in Table 2 and illustrated in Figure 2; ROC curves are shown in Figure 3.

#### Secondary Analysis: Model Performance Without IL-6

When IL-6 was excluded, XGBoost achieved an AUC of 0.86 (95% CI: 0.83–0.89), with a sensitivity of 0.81, specificity of 0.79, PPV of 0.70, NPV of 0.88, and an F1-score of 0.75—a modest but clinically acceptable reduction compared to the full model (AUC 0.89). SHAP rankings were preserved: serum lactate, free intraperitoneal air, and bowel ischemia remained dominant contributors, confirming strong retained performance in resource-limited settings where IL-6 is unavailable.

### 3.5. Confusion Matrix and Classification Performance

At the optimal probability threshold (Youden index), the XGBoost model demonstrated 262 true positives, 138 false positives, 582 true negatives, and 50 false negatives. This corresponds to an accuracy of 0.82, a sensitivity of 0.84, and a specificity of 0.81, indicating strong ability to correctly identify patients requiring surgery while maintaining an acceptable false-positive rate.

### 3.6. Calibration and Model Reliability

Calibration analysis demonstrated good agreement between predicted and observed probabilities for all three models. For the XGBoost model, Hosmer–Lemeshow test *p* = 0.41; calibration slope = 0.97; calibration intercept = 0.02. Calibration plots for Random Forest and logistic regression showed slightly wider confidence bands but remained within acceptable limits.

**Figure 4:**
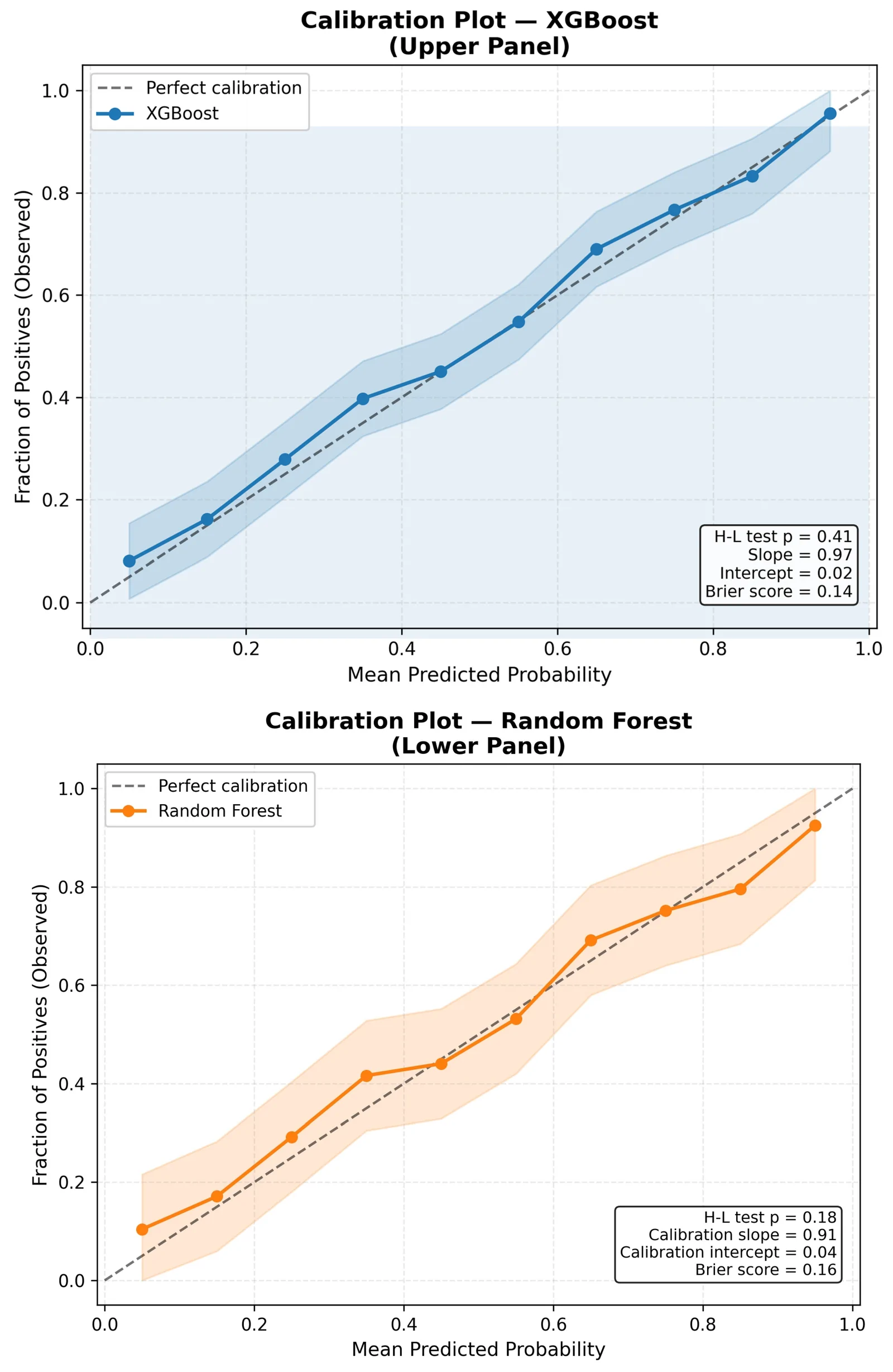
Calibration plots for XGBoost (**upper panel**) and Random Forest (**lower panel**), demonstrating strong agreement between predicted probabilities and observed outcomes across risk strata.

The Brier score for XGBoost was 0.14 (Random Forest: 0.16; logistic regression: 0.19), confirming superior probabilistic accuracy. Calibration plots for XGBoost and Random Forest are presented in Figure 4.

### 3.7. Decision Curve Analysis

**Figure 5:**
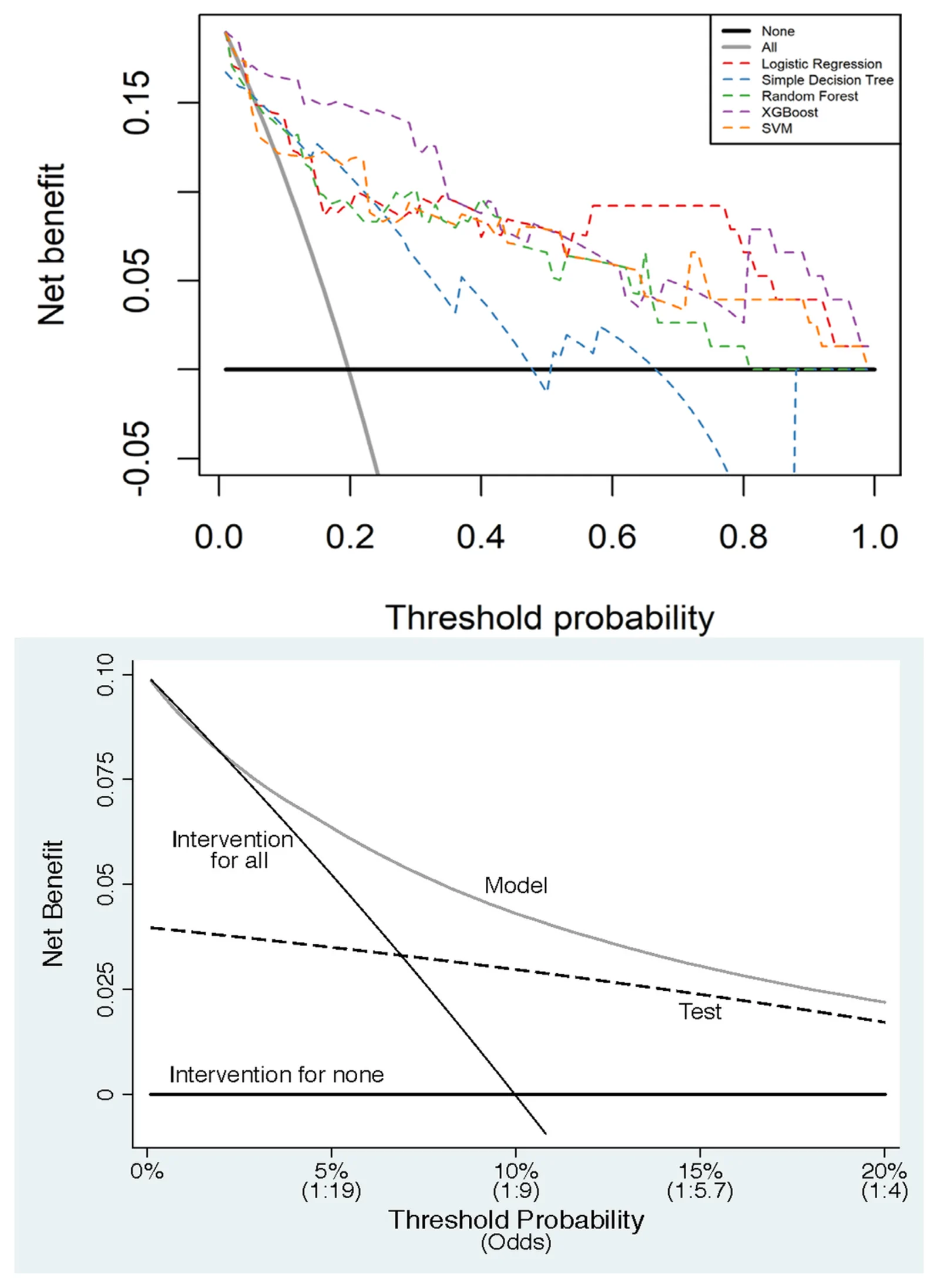
Decision curve analysis for XGBoost (**upper panel**) and Random Forest (**lower panel**), demonstrating net clinical benefit across a wide range of threshold probabilities compared to treat-all and treat-none strategies.

Decision curve analysis demonstrated that the XGBoost model provides net clinical benefit across threshold probabilities between 10% and 70%, outperforming both ‘treat-all’ and ‘treat-none’ strategies. This supports the model’s applicability in real-world decision-making, particularly in intermediate-risk patients. Decision curves for XGBoost and Random Forest are shown in Figure 5.

### 3.8. Comparison with Traditional Clinical Scores

When compared to established clinical scoring systems applied to the same cohort, the XGBoost model significantly outperformed all traditional approaches, particularly due to its integration of imaging findings and early biomarkers (Table 3).

### 3.9. Multivariable Predictors of Surgical Intervention

Multivariable logistic regression confirmed that the following variables independently predicted surgical necessity (Table 4). These findings confirm that surgical necessity is driven by a combination of systemic and structural pathology.

### 3.10. Explainability Analysis

**Figure 6:**
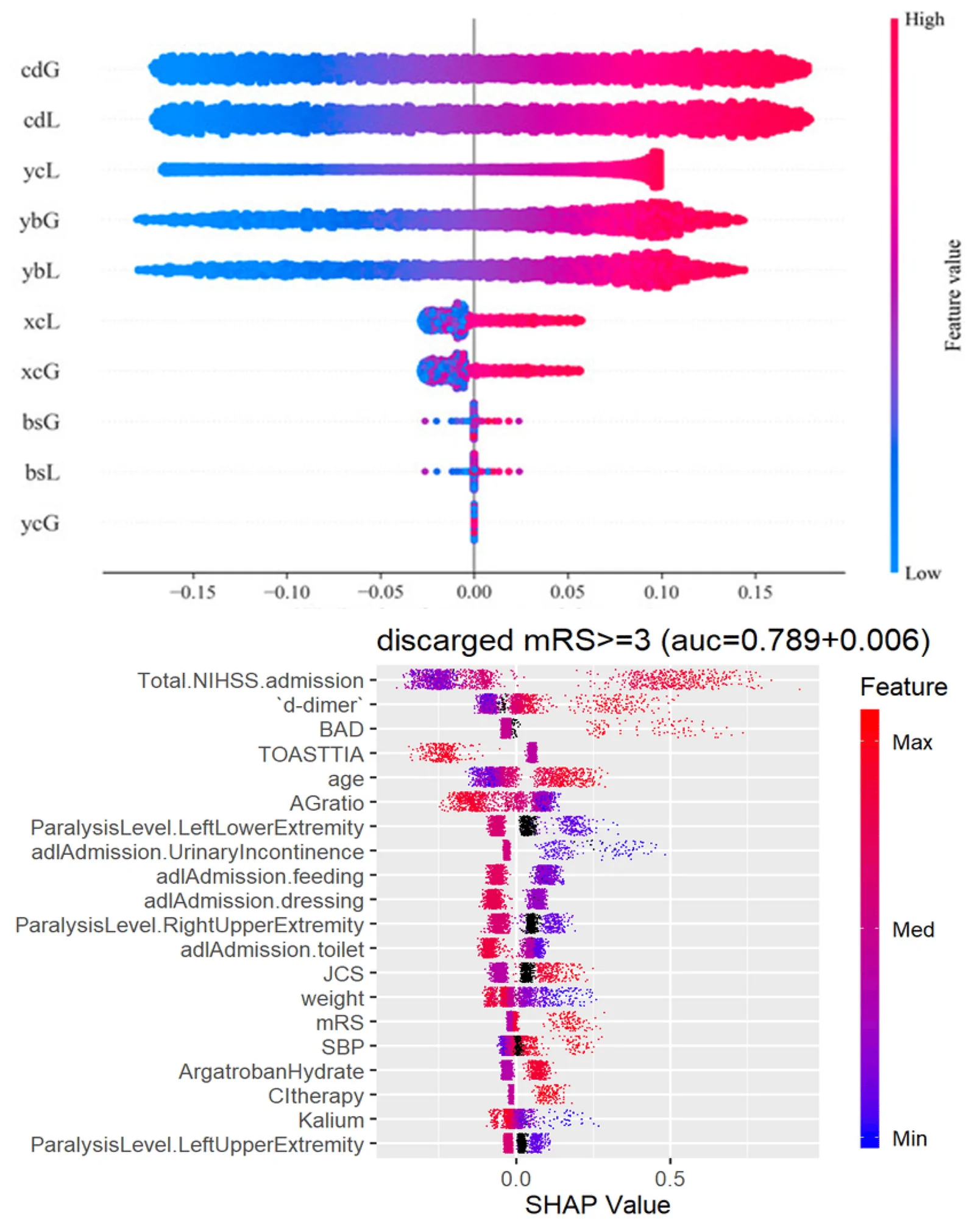
Global SHAP summary plot (**upper panel**) showing feature importance and directionality across the cohort. Individual SHAP force plot (**lower panel**) illustrating patient-level prediction decomposition for a representative high-risk case.

SHAP analysis demonstrated that lactate, imaging findings (free intraperitoneal air and bowel ischemia), IL-6, and shock index were the most influential predictors at the cohort level. Higher values of lactate and inflammatory markers consistently shifted predictions toward surgical intervention. At the individual level, SHAP force plots confirmed that predictions were driven by combinations of variables rather than single factors, particularly in borderline clinical scenarios. Global feature importance and an individual-level SHAP force plot are presented in Figure 6.

### 3.11. Subgroup Analysis

Model performance remained consistent across major diagnostic categories, supporting generalizability across the heterogeneous spectrum of GI emergencies (Table 5).

### 3.12. Secondary Outcomes

Patients requiring surgical intervention had significantly worse secondary outcomes compared with those managed conservatively:

1. In-hospital mortality: 14.1% vs. 4.3% (*p* < 0.001)
2. Length of hospital stay: 14 days vs. 7 days (*p* < 0.001)
3. ICU admission rate: 38.5% vs. 12.2% (*p* < 0.001)

### 3.13. Integrated Interpretation

The results demonstrate that early prediction of surgical necessity in gastrointestinal emergencies is both feasible and clinically meaningful. Machine learning models significantly improve predictive accuracy by capturing complex, non-linear relationships between clinical, laboratory, and imaging variables. The integration of SHAP-based explainability ensures transparency, supporting the implementation of explainable machine learning as a decision-support tool for early triage.

However, a critical interpretation of the PPV is essential: a PPV of 0.74 implies that approximately one in four patients flagged as requiring surgery may not need operative intervention, while false negatives remain a clinically serious concern. The model should not be presented as a high-accuracy standalone tool. Rather, it is best understood as a decision-support adjunct that augments—but does not replace—experienced surgical judgment, with greatest utility in settings where immediate senior surgical expertise is limited.

## 4. Discussion

The present study demonstrates that explainable machine learning can accurately and transparently predict the need for surgical intervention in patients presenting with gastrointestinal emergencies using routinely available early clinical, laboratory, and imaging data. The principal findings are that (i) machine learning models, particularly XGBoost, significantly outperform traditional logistic regression; (ii) a consistent predictive triad—metabolic (lactate), inflammatory (IL-6), and structural (imaging findings)—underlies early surgical necessity; and (iii) SHAP-based explainability provides clinically meaningful insight, supporting real-world applicability.

### 4.1. Interpretation of the Main Findings

A key contribution of this study is the identification of a pathophysiological convergence early in the disease course, where metabolic stress, systemic inflammation, and structural pathology collectively determine the likelihood of surgical intervention. Serum lactate emerged as the strongest predictor, reflecting early microcirculatory dysfunction and tissue hypoxia, and its elevation was observed even in the absence of overt hemodynamic instability. IL-6 captured the initial inflammatory cascade not adequately reflected by traditional markers such as CRP. Imaging findings, particularly free intraperitoneal air and bowel ischemia, remained strong independent predictors; however, the added value of the ML approach lies in integrating these findings with systemic physiological signals, allowing a more nuanced and dynamic assessment. Importantly, the model demonstrated superior performance in borderline cases where traditional decision-making is most uncertain.

### 4.2. Comparison with Existing Literature

Traditional scoring systems such as APACHE II, SOFA, and BISAP are primarily designed to predict severity or mortality rather than surgical necessity, and their performance is limited by the inability to incorporate imaging findings and complex variable interactions. The recent systematic review by Critelli et al. [12] highlighted both the rapid proliferation of ML prognostic models in acute pancreatitis and persistent gaps in methodological rigor—limitations the present study addresses through structured cross-validation, calibration assessment, and TRIPOD+AI-compliant reporting. The ML-based emergency surgery risk calculator reported by Chen et al. [10] focused on postoperative outcomes rather than the pre-operative decision of whether surgery is required—a clinically upstream and arguably more consequential question that this study directly addresses. CT-based radiomics models [13] and clinical-radiomics nomograms for infected pancreatic necrosis [14] demonstrated the feasibility of quantitative imaging biomarkers; the present study extends this by integrating radiological findings with metabolic and inflammatory markers across a broad spectrum of GI emergencies.

### 4.3. Clinical Implications and Practical Utility

The findings of this study support a transition from reactive, deterioration-based decision-making toward a predictive and proactive approach. By identifying early markers—specifically lactate, IL-6, and imaging findings—the model enables clinicians to recognize high-risk patients before clinical deterioration becomes evident. This has direct implications for patients in the diagnostic gray zone, where the current standard is often a ‘wait-and-see’ strategy. The model provides a means to objectify risk in borderline scenarios, facilitating earlier escalation of care, including intensified monitoring, earlier repeat imaging, or preemptive surgical consultation.

Lactate, traditionally interpreted as a marker of shock or late-stage sepsis, is shown here to be relevant even at moderate elevations when interpreted in combination with other variables. Similarly, IL-6 provides insight into the early inflammatory trajectory. Imaging interpretation also benefits from this integrative approach: subtle findings such as minimal bowel wall thickening may acquire clinical significance when associated with metabolic and inflammatory abnormalities. From an operational perspective, this approach may improve workflow through earlier prioritization of high-risk patients, more efficient ICU allocation, and timely multidisciplinary discussions.

The applicability of this model must be considered in the context of healthcare system variability. The inclusion of IL-6, while valuable, represents a limitation in settings where it is costly or unavailable. Access to early contrast-enhanced CT and computational infrastructure may not be universal. These findings ultimately support a transition toward data-augmented clinical decision-making, where ML tools enhance rather than replace clinical expertise.

### 4.4. Strengths and Limitations

This study benefits from a large, heterogeneous cohort representative of real-world emergency practice across a six-year period. The integration of multimodal data, rigorous cross-validation, and SHAP-based explainability represent major strengths. Compliance with STROBE and TRIPOD+AI reporting standards supports reproducibility and transparent appraisal.

Several limitations must be acknowledged. First, the retrospective single-center design limits generalizability, and external validation in independent multicenter cohorts is required. Second, IL-6 was available in 71.8% of patients; although multiple imputation was applied, this may affect reproducibility in resource-limited settings. Third, imaging interpretation by blinded radiologists may still introduce inter-observer variability. Fourth, the binary endpoint of surgical intervention may be influenced by institutional practices and evolving non-operative management strategies. Finally, Youden index threshold selection may not be optimal for all clinical contexts; threshold optimization should be explored in future prospective studies.

Fifth, the PPV of 0.74 implies that approximately one in four patients flagged as requiring surgery may not actually need operative intervention, precluding use of this model as a standalone surgical decision tool. Performance may vary across institutions with different case-mix distributions, IL-6 assay platforms, and surgical decision cultures. External validation across at least three to five diverse centers is required before deployment.

To further address this limitation, we propose a specific external validation framework: a prospective multicenter study enrolling at least 500 patients across three to five independent tertiary emergency centers, with pre-specified performance benchmarks (non-inferiority AUC threshold 0.83) and stratified analysis by diagnostic subgroup, healthcare setting, and IL-6 availability. Until such validation is completed, the model should be considered hypothesis-generating and used only as a supplementary triage tool under direct clinician supervision.

Sixth, the Youden index does not reflect the asymmetric cost of emergency surgical decision-making, where the cost of a missed surgical indication substantially exceeds that of an unnecessary consultation. Sensitivity-weighted threshold optimization is recommended for prospective implementation studies.

### 4.5. Conclusions

This study demonstrates that explainable machine learning can accurately predict early surgical necessity in gastrointestinal emergencies by integrating metabolic, inflammatory, and imaging data available within the first 24 h of presentation. The XGBoost model significantly outperformed traditional scoring systems (AUC 0.89, NPV 0.90), supporting its utility for both risk escalation and conservative management decisions. SHAP-based explainability provides the transparency necessary for clinical adoption. Future multicenter, prospective studies are needed to validate these findings and facilitate integration into routine clinical practice.

However, the PPV of 0.74—implying one in four flagged patients may not require surgery, with false negatives remaining a critical concern—precludes positioning this model as a standalone surgical decision tool. The model is best understood as a decision-support adjunct, augmenting experienced surgical judgment particularly in settings where senior expertise is not immediately available. Prospective multicenter validation is a prerequisite before integration into routine practice can be recommended.

The absence of external validation on an independent cohort represents the most critical limitation of the present work and must be explicitly framed as the primary direction for future research. The model’s real-world applicability and generalizability cannot be established without this step, and no recommendation for clinical deployment should be made prior to prospective multicenter validation.

## Figures and Tables

**Table 1:** Baseline characteristics of the overall cohort and by surgical outcome.

Variable	Overall (n = 1032)	Surgery (n = 312)	No Surgery (n = 720)	*p*-Value
Demographics and Clinical Parameters				
Age (years)	64 (52–75)	66 (54–77)	62 (51–73)	0.021
Male sex, n (%)	601 (58.2)	198 (63.5)	403 (56.0)	0.028
BMI (kg/m^2^)	27.1 ± 4.3	27.6 ± 4.5	26.9 ± 4.2	0.074
ASA ≥ III, n (%)	512 (49.6)	182 (58.3)	330 (45.8)	<0.001
Shock index > 0.9, n (%)	284 (27.5)	132 (42.3)	152 (21.1)	<0.001
Laboratory Parameters (first 24 h)				
WBC (×10^9^/L)	13.2 (10.1–17.4)	15.8 (12.6–19.3)	12.4 (9.8–15.6)	<0.001
CRP (mg/L)	118 (65–210)	186 (120–280)	94 (52–165)	<0.001
IL-6 (pg/mL) *	72 (38–150)	132 (78–240)	54 (30–102)	<0.001
Lactate (mmol/L)	2.1 (1.4–3.6)	3.2 (2.1–5.1)	1.8 (1.2–2.8)	<0.001
Creatinine (mg/dL)	1.18 (0.9–1.6)	1.42 (1.1–1.9)	1.05 (0.8–1.4)	<0.001
Hemoglobin (g/dL)	12.8 ± 2.1	12.2 ± 2.3	13.1 ± 1.9	0.002
Imaging Findings (CECT)				
Free intraperitoneal air, n (%)	196 (19.0)	138 (44.2)	58 (8.1)	<0.001
Bowel ischemia signs, n (%)	148 (14.3)	102 (32.7)	46 (6.4)	<0.001
Bowel wall thickening, n (%)	372 (36.0)	162 (51.9)	210 (29.2)	<0.001
Reduced bowel enhancement, n (%)	128 (12.4)	88 (28.2)	40 (5.6)	<0.001
Intra-abdominal fluid collections, n (%)	472 (45.7)	188 (60.3)	284 (39.4)	<0.001
Pancreatic necrosis (pancreatitis subset), n (%)	78/352 (22.2)	54/352 (15.3)	24/352 (6.8)	<0.001

* IL-6 was available in 741/1032 patients (71.8%); missing values were imputed using multiple imputation with chained equations. Continuous variables: mean ± SD or median (IQR). Categorical variables: n (%). Abbreviations: BMI, body mass index; ASA, American Society of Anesthesiologists; WBC, white blood cell count; CRP, C-reactive protein; IL-6, interleukin-6; CECT, contrast-enhanced computed tomography.

**Table 2:** Comparative performance of predictive models on the test cohort.

Model	AUC (95% CI)	Sensitivity	Specificity	PPV	NPV	F1-Score
Logistic Regression	0.79 (0.76–0.82)	0.71	0.73	0.61	0.81	0.66
Random Forest	0.86 (0.83–0.89)	0.80	0.78	0.70	0.87	0.75
**XGBoost**	**0.89 (0.86–0.92)**	**0.84**	**0.81**	**0.74**	**0.90**	**0.79**

AUC, area under the receiver operating characteristic curve; CI, confidence interval; PPV, positive predictive value; NPV, negative predictive value. Threshold determined by the Youden index. XGBoost results in bold.

**Table 3:** Comparison of the XGBoost model with traditional clinical scoring systems.

Score	AUC
APACHE II	0.72
SOFA	0.74
BISAP (pancreatitis subset)	0.76
XGBoost (current model)	0.89

BISAP comparison restricted to the acute pancreatitis subgroup (n = 352). AUC, area under the receiver operating characteristic curve.

**Table 4:** Independent multivariable predictors of surgical intervention.

Variable	OR	95% CI	*p*-Value
Free intraperitoneal air	4.2	2.9–6.1	<0.001
CT evidence of ischemia	3.8	2.6–5.5	<0.001
Lactate (mmol/L)	2.4	1.9–3.1	<0.001
IL-6 (pg/mL)	1.8	1.3–2.4	0.002
Shock index	1.6	1.2–2.1	0.010

OR, odds ratio; CI, confidence interval. For all variables, *p* < 0.05 after adjustment for age, sex, BMI, and ASA status.

**Table 5:** XGBoost model performance by diagnostic subgroup.

Subgroup	AUC (XGBoost)
Acute pancreatitis	0.87
Bowel obstruction	0.88
Perforation	0.91
Mesenteric ischemia	0.90

AUC, area under the receiver operating characteristic curve.

## Data Availability

The data supporting the findings of this study are available from the corresponding author upon reasonable request, subject to institutional data governance regulations and patient privacy requirements.

## Abbreviations

The following abbreviations are used in this manuscript:

AI	artificial intelligence
APACHE II	Acute Physiology and Chronic Health Evaluation II
ASA	American Society of Anesthesiologists
AUC	area under the receiver operating characteristic curve
BISAP	Bedside Index of Severity in Acute Pancreatitis
BMI	body mass index
CECT	contrast-enhanced computed tomography
CI	confidence interval
CRP	C-reactive protein
DCA	decision curve analysis
GI	gastrointestinal
ICU	intensive care unit
IL-6	interleukin-6
IQR	interquartile range
MDT	multidisciplinary team
MICE	multiple imputation by chained equations
ML	machine learning
NPV	negative predictive value
OR	odds ratio
PPV	positive predictive value
ROC	receiver operating characteristic
SD	standard deviation
SHAP	Shapley Additive Explanations
SOFA	Sequential Organ Failure Assessment
STROBE	Strengthening the Reporting of Observational Studies in Epidemiology
TRIPOD+AI	Transparent Reporting of a Multivariable Prediction Model—Artificial Intelligence
WBC	white blood cell count
XAI	explainable artificial intelligence
XGBoost	Extreme Gradient Boosting
